# Supplementary material for: The antioxidant effects of butylated hydroxytoluene on cryopreserved goat sperm from a proteomic perspective
Source: PeerJ. 2024 Jul 5;12:e17580. doi: 10.7717/peerj.17580 (PMC11229688; doi:10.7717/peerj.17580)
Supplement: Supplemental Information 1 [file peerj-12-17580-s001.pdf]

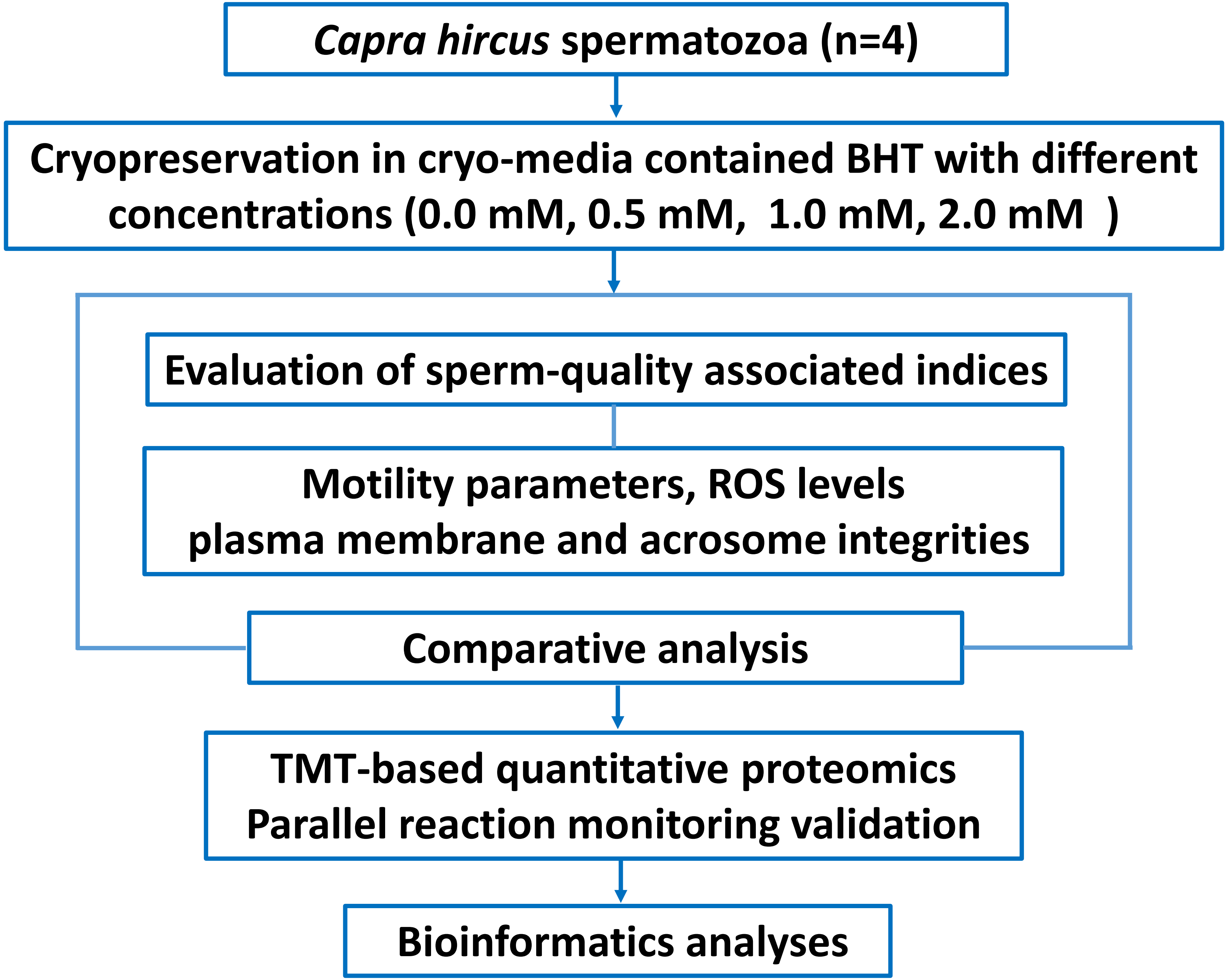
*Capra hircus* spermatozoa (n=4)
Cryopreservation in cryo-media contained BHT with different concentrations (0.0 mM, 0.5 mM, 1.0 mM, 2.0 mM )
Evaluation of sperm-quality associated indices
Motility parameters, ROS levels
plasma membrane and acrosome integrities
Comparative analysis
TMT-based quantitative proteomics
Parallel reaction monitoring validation
Bioinformatics analyses
