# Supplementary figures and images for: The antioxidant effects of butylated hydroxytoluene on cryopreserved goat sperm from a proteomic perspective
Source: PeerJ. 2024 Jul 5;12:e17580. doi: 10.7717/peerj.17580 (PMC11229688; doi:10.7717/peerj.17580)

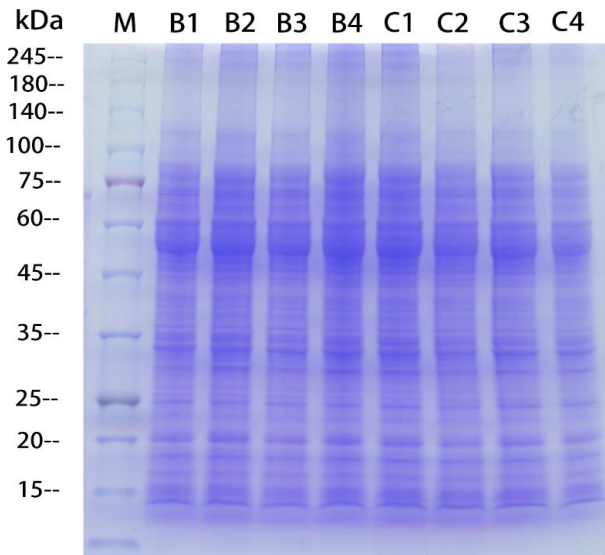

Supplement: Supplemental Information 2 [file peerj-12-17580-s002.pdf]
